# Sensor Modality Versus Pretraining for Recognising Bed-Exit–Relevant Postural Transitions: A Gyroscope-Focused Feasibility Study on Public Wearable-IMU Data

**DOI:** 10.3390/s26196073

**Published:** 2026-09-25

**Authors:** Youmi Park, Bayarbaatar Bold, Wonchul Cha

**Affiliations:** 1 Office of Management and Support, Heart, Vascular and Stroke Institute (HVSI), Samsung Medical Center, Seoul 06351, Republic of Korea; youmi.park@samsung.com; 2 Department of Digital Health, Samsung Advanced Institute for Health Sciences and Technology (SAIHST), Sungkyunkwan University, Seoul 06355, Republic of Korea; 3 Healthcare Digital Transformation Research Institute, Samsung Advanced Institute for Health Sciences and Technology (SAIHST), Sungkyunkwan University, Seoul 06355, Republic of Korea; bayarbaatar@skku.edu; 4 Department of Emergency Medicine, Samsung Medical Center, Seoul 06351, Republic of Korea

**Keywords:** human activity recognition, wearable inertial measurement unit (IMU), gyroscope, motion foundation model, postural transition, bed-exit monitoring, inpatient falls

## Abstract

Wearable ward monitoring must eventually detect when a patient leaves the bed, yet the brief postural transitions that begin a bed exit are under-examined relative to dynamic activities. As a feasibility study, we used a public waist-worn six-axis inertial dataset (30 healthy subjects) to ask which sensor axes carry these transitions and whether a pretrained foundation model helps. We defined a ten-class task of four basic activities and six directional transitions, treating the two lie-to-upright transitions as the bed-exit-relevant classes, evaluated with subject-grouped cross-validation. Postural transitions were the weakest classes, and the weakest of these was a bed-exit transition. Removing the gyroscope reduced macro-F1 from 0.822 to 0.647 and bed-exit F1 from 0.657 to 0.351 (*p* < 10^−4^) while leaving lying and walking unaffected. The mirror ablation separated the roles: the accelerometer captured posture and the gyroscope captured every directional transition. A six-axis hand-crafted model outperformed one accelerometer-only foundation model even after full fine-tuning (0.822 vs. 0.774) with far fewer labels. Scoring windows that cross bout boundaries lowered bed-exit sensitivity from 0.937 to 0.833. Among the configurations tested, sensor choice, the gyroscope in particular, mattered more than model scale. These results concern transition recognition in scripted recordings, not bed-exit detection on a ward.

## 1. Introduction

Falls are among the most frequently reported adverse events in hospital inpatients, and a substantial minority result in injury, prolonged admission, or loss of independence at discharge [1]. Their occurrence is not uniformly distributed across admission. In a prospective series of 183 inpatient falls, 79% were unassisted, 85% occurred in the patient’s own room, and 59% occurred during the evening or overnight. Half were elimination-related; that is, they occurred while the patient was attempting to reach a toilet or commode [2]. These findings identify bed-exit initiation, particularly the transition from lying to sitting or standing, as a potentially actionable point for monitoring. A system that detects this transition in real time could trigger assistance before the patient begins unsupported ambulation. Although timely detection alone cannot prevent a fall, it may create an opportunity for earlier intervention than systems designed primarily to detect or record the fall itself.

Acting on that transition requires recognising what the patient is doing on the bed. The decisive distinction is not between movement and stillness, since a patient in bed moves constantly. It is between behaviour that keeps the patient on the bed, whether lying still, turning, or settling back down, and the movement that carries the patient off it. Only the latter precedes a fall, and the two are separated by the direction of the postural change and the body configuration it ends in, rather than by the amount of motion involved. This is where sensors that register load rather than behaviour fail. A pressure pad or a weight-sensing mattress reports that the load on the bed has changed, not which of these movements produced the change, so repositioning and a genuine egress are read alike. The clinical consequence is visible in the trial record. A cluster randomised trial that raised bed-alarm use thirty-fold found no reduction in falls, injurious falls, or physical restraint use [3]. A subsequent disinvestment trial found that reducing alarm use was non-inferior to usual care while lowering patient-reported sleep disturbance [4]. Systems that do attempt to anticipate a bed exit accordingly move from load to behaviour, predicting egress from estimated patient posture [5] or from the in-bed pressure-distribution pattern that precedes it [6]. The recognition target for a ward monitor is therefore behavioural: the postures a patient holds in bed, and the directional transition that takes them out of it.

Body-worn inertial sensing is the plausible route to that behavioural recognition and is already entering wards for mobility tracking and fall detection [7,8,9,10]. Wearable systems aimed specifically at bed exit already exist and establish their feasibility. They include a dedicated low-power inertial node for bed-exit signalling on a geriatric ward [11], a battery-less radio-frequency identification (RFID)-plus-accelerometer tag evaluated in hospitalised older patients [12], and a gyroscope-bearing inertial unit that recognises the lying-to-sitting transition within a bedtime-monitoring system [13]. These works show that a wearable can flag a bed exit. Each, however, treats the device as a fixed system rather than asking which sensor axes the transition requires, whether a pretrained representation helps, or how much labelled observation the task demands. Those are questions a reproducible public benchmark can settle before a device is committed. Two clinical requirements separate this application from consumer activity tracking. First, the target is a brief transition rather than a sustained activity. Rising from bed takes only a few seconds and occurs a handful of times a day, so a system tuned to sustained walking will under-serve it. Second, the direction of the transition carries the clinical meaning. Lying-to-sitting and sitting-to-lying produce superficially similar signals, but only the first indicates a patient leaving the bed, and collapsing them discards precisely the information a ward monitor must act on.

Human activity recognition (HAR) from inertial sensors is nonetheless often described as a solved problem, with reported accuracies above 95% on standard benchmarks [14,15]. Those benchmarks are dominated by whole-body dynamic activities such as walking and stair climbing, which are easy to separate and are largely absent from a ward. The brief postural transitions that matter clinically, and the distinction between low-motion postures such as sitting and standing, are comparatively under-examined [16]. Where transitions have been treated as a target in their own right, the work has concentrated on raising recognition accuracy with richer architectures [17] rather than on asking which sensor channels the transitions require. In parallel, the field has produced large motion foundation models pretrained on millions of hours of wearable data [18,19,20,21,22], raising the expectation that a pretrained representation should outperform task-specific models. These models are trained predominantly on accelerometer corpora, a design choice whose consequences for transition recognition have not been examined. Because rising from bed is mechanically a rotation of the trunk, the presence of an angular-rate channel is a plausible determinant of whether the event can be recognised at all. Yet sensor configuration is rarely treated as a study variable alongside model choice.

These strands leave three questions unanswered for a ward-monitoring application. How well does a body-worn inertial unit recognise ward-relevant postural transitions, as opposed to easy dynamic activities? Which sensor axes carry the information those transitions require? And does a pretrained motion foundation model improve on a simple task-specific model for this task? A fourth, operational question follows from them, namely, how much labelled bedside observation a prospective ward study would have to fund in order to learn these events. Together, these determine whether design effort is better spent on model scale or on sensor selection. That trade-off has not been tested for ward-relevant transitions on data in which both accelerometer and gyroscope are available.

We addressed these questions on a public waist-worn six-axis inertial dataset as a feasibility study for a planned prospective ward observation study. We defined a ward-relevant recognition task centred on directional postural transitions and bed-exit events. We quantified the contribution of the gyroscope by ablation against the same model with the gyroscope removed, measured the labelling requirement, and compared hand-crafted representations against a pretrained motion foundation model under an identical subject-grouped protocol. Results obtained on healthy volunteers performing scripted movements are treated throughout as an upper bound on ward performance, and are used to specify the sensor configuration, labelling budget, and hypotheses of the subsequent clinical study.

The objectives of this study were therefore:

1. To quantify how well a waist-worn inertial unit recognises ward-relevant postural transitions, and bed-exit transitions in particular, relative to the basic activities that dominate aggregate recognition scores;
2. To determine which sensor axes carry that information, by ablating the gyroscope and, as its mirror, the accelerometer, so that the contribution of gravity and of rotation can be separated rather than only removed;
3. To test whether a pretrained accelerometer-only motion foundation model improves on a simple task-specific model under an identical subject-grouped protocol, and whether any difference reflects model scale or the sensor channels available to the model;
4. To estimate the labelling requirement, that is, how much annotated observation a prospective ward study must fund, and to express it in the units such a study would plan with;
5. To characterise what these estimates would cost in deployment, by scoring the same models as a bed-exit alarm across probability thresholds and on windows that cross activity boundaries, as a continuously sliding window must.

## 2. Materials and Methods

### 2.1. Study Design

This was a feasibility and benchmarking study conducted entirely on a public, de-identified dataset, designed to inform the sensor configuration, labelling budget, and analysis plan of a subsequent prospective ward-based observation study. It addressed the five objectives set out at the end of the Introduction.

The design has four stages, summarised in Figure 1. First, the public Human Activities and Postural Transitions (HAPT) dataset (30 subjects wearing a waist-mounted smartphone whose triaxial accelerometer and gyroscope were sampled at 50 Hz) was segmented into 2.56 s windows with 50% overlap, each placed strictly inside a single annotated activity bout. The windows were mapped onto a ten-class ward-relevant proxy task of four basic activities and six directional postural transitions, of which the two lie-to-upright transitions were designated bed-exit events. Second, the representations to be compared were computed on those same windows. Hand-crafted feature sets were built from all six axes, from the three accelerometer axes alone, and from the three gyroscope axes alone. They were set against the accelerometer-only UniMTS foundation model read out three ways: as a frozen linear probe, as a frozen embedding classified by gradient-boosted trees, and with full fine-tuning. The available input differs between these arms by construction rather than by choice: the released foundation model accepts accelerometer channels only. Third, every arm was evaluated under an identical protocol, subject-grouped five-fold cross-validation with the subject as the unit of analysis. Each was reported as macro-F1, bed-exit F1, and label efficiency against the annotated minutes available for training. Fourth, the deployable six-axis configuration was scored as a bed-exit alarm, across probability thresholds and at the level of whole events, and re-scored on windows slid across activity boundaries, as a continuously sliding window must be.

Results on healthy volunteers performing scripted activities are treated throughout as an upper bound on ward performance, with the domain gap addressed in Section 4.

### 2.2. Datasets

The Smartphone-Based Recognition of Human Activities and Postural Transitions (HAPT) dataset [23] was used throughout. Its 30 volunteers (aged 19–48 years) wore a waist-mounted smartphone whose triaxial accelerometer and triaxial gyroscope were recorded at 50 Hz, yielding a six-axis inertial signal. The acquisition device and its sensor specifications are summarised in Table 1. HAPT labels six basic activities (walking, walking upstairs, walking downstairs, sitting, standing, lying) and, uniquely among common HAR benchmarks, six directional postural transitions (stand-to-sit, sit-to-stand, sit-to-lie, lie-to-sit, stand-to-lie, lie-to-stand). This label set is the reason the dataset was chosen. It is one of the very few widely used public corpora that both preserve transition direction and record the gyroscope alongside the accelerometer, which is what makes the sensor-configuration question answerable.

No a priori sample-size calculation was performed. All available subjects in the public dataset were used, and the label-efficiency analysis (Section 3.4) characterises the data requirement empirically in place of a power calculation. The dataset is complete, and the only records excluded were activity segments shorter than a single analysis window, reported in the Results.

### 2.3. Ward-Relevant Task and Windowing

We defined a ten-class ward-relevant recognition task comprising four basic activities encountered in a ward (lying, sitting, standing, walking) and the six directional postural transitions. Stair climbing and descent were excluded because they do not occur in a typical ward and would inflate aggregate accuracy. The full twelve-class list and its mapping to the ten-class ward task is given in Table S1. The two lie-to-upright transitions (lie-to-sit and lie-to-stand) were designated bed-exit events, as they correspond to a patient leaving the bed and are the transitions most relevant to fall risk [9].

To show what the ward-relevant restriction costs and where a conventional benchmark score comes from, the standard twelve-class HAPT problem was also run with the same six-axis model, features, and cross-validation protocol. That problem adds stair ascent and descent to the ten ward classes. It serves only as a reference point for decomposing an aggregate score by class and is not used for any comparative inference.

The operational contrast this task can support should be stated precisely. HAPT labels lying as a single static posture and does not annotate movements made while the subject remains recumbent, such as turning or repositioning. The static lying class therefore stands as a proxy for remaining on the bed. The discrimination actually evaluated here is between that state, the transitions that return a subject to it (sit-to-lie, stand-to-lie), and the transitions that leave it (lie-to-sit, lie-to-stand). Separating true in-bed repositioning from the onset of a bed exit requires labels that no public inertial corpus provides and is deferred to the prospective ward study, in which an observer annotates behaviour at the bedside.

Continuous inertial recordings were segmented into fixed-length windows placed strictly within a single labelled activity bout, so that no window straddled two activities or crossed a subject boundary. The primary analysis used 2.56 s windows (128 samples at 50 Hz), the established convention for this data family, with 50% overlap [24]. Because postural transitions are brief, and several are shorter than 2.56 s, we additionally swept window lengths from 0.64 to 2.56 s to quantify the trade-off between temporal coverage of short transitions and per-window information. This sweep was repeated on the subset of segments long enough to yield a window at every length, to remove a survivorship confound.

### 2.4. Representations and Models

We compared hand-crafted feature representations against a pretrained motion foundation model, holding the downstream classifier and the evaluation protocol fixed so that only the representation differed.

*Preprocessing: none, and why.* No filtering, detrending, or per-recording normalisation was applied, and the gravitational component of the acceleration signal was deliberately retained. Signals were used in the units supplied by the dataset. This is a deliberate design decision rather than an omission. Gravity determines how acceleration is distributed across the three axes at rest, so it is the signal that separates lying from sitting and standing, the low-motion classes of central interest here. In the raw recordings, the gravity vector loads the y-axis while a subject is lying (mean +0.95 g over a 3 s excerpt) and the x-axis while sitting or standing (+1.02 g and +0.96 g). Sitting and standing are separated mainly by the y component (−0.03 g against −0.39 g). The gyroscope meanwhile stays within ±0.08 rad/s while a posture is held and reaches 1.91 rad/s during a bed-exit transition. A high-pass filter, detrending, or per-recording normalisation would remove precisely the constant offsets that encode posture. Figure S2 in the Supplementary Materials shows these raw six-axis traces for three static postures and one bed-exit transition. Because the 50% window overlap operates within a subject and all splits are made by subject, overlapping windows are never divided between a training and a test fold.

*Hand-crafted features.* From each window, we extracted a standard set of 18 time-domain and frequency-domain descriptors per channel [25]: mean, standard deviation, extrema, median, interquartile range, mean absolute deviation, energy, root mean square, skewness, kurtosis, zero-crossing rate, jerk statistics, dominant frequency, spectral centroid, spectral entropy, and spectral energy. In plain terms, these are numerical summaries of how much, how fast, and how rhythmically the signal moves. The frequency-domain descriptors separate the regular rhythm of walking from the single aperiodic sweep of a postural change, while the accelerometer inter-axis correlations encode how gravity is distributed across the axes and therefore posture. Descriptors were computed for each sensor axis and for the vector magnitude of each sensor. Combining accelerometer and gyroscope channels is standard practice in inertial activity recognition [26]. To isolate the contribution of each sensor, features were computed in three configurations: a six-axis accelerometer-plus-gyroscope set of 150 features, a three-axis accelerometer-only set of 75, and a three-axis gyroscope-only set of 75. Each three-axis set was recomputed from its own channels alone rather than obtained by deleting columns from the six-axis set. Each configuration comprises the 18 descriptors for every channel, that is, the individual axes and each sensor’s vector magnitude, together with the three inter-axis correlations of each sensor. This gives eight channels in the six-axis case and four in either three-axis case, which is how the counts of 150 and 75 arise (18 × 8 + 6 and 18 × 4 + 3). All three were classified with gradient-boosted trees (XGBoost) [27] using a fixed configuration (400 trees, maximum depth 6, learning rate 0.08, subsample and column-subsample 0.8). These values were fixed in advance at conventional settings and were not tuned on these data. They were applied unchanged to every representation, including the foundation-model embeddings, so that no comparison reflects a difference in tuning effort rather than in the representation itself. The classifier is held fixed by design: the study compares what a representation makes available, not which learner extracts it best, and allowing the classifier to vary alongside the representation would confound the two. This design is only informative if the resulting ordering does not depend on the learner. We therefore verified that separately by repeating the representation comparison under a random forest and a scaled multinomial logistic regression on the same folds, with the scaler fitted inside each training fold. The result is reported in Section 3.5. No class weighting or resampling was applied to the gradient-boosted models. The rare transition classes were instead protected by the choice of macro-F1 as the primary metric, which weights every class equally regardless of its frequency. The three-axis accelerometer-only configuration takes the same input as the released foundation model and therefore serves as the like-for-like baseline that isolates the contribution of large-scale pretraining.

*Foundation model.* We used UniMTS [21], a motion time-series foundation model that aligns a spatio-temporal graph-convolutional encoder [28] over a 22-joint skeleton with a contrastive language embedding [29]. In plain terms, UniMTS is a large model pretrained on very large amounts of movement data. It belongs to the same broad family as the large language models now familiar in medicine but is built for body motion rather than text. The publicly released checkpoint was obtained from its Hugging Face repository (https://huggingface.co/xiyuanz/UniMTS, accessed on 17 July 2026). HAPT is not part of its pretraining corpus, so the evaluation data are held out from the model. The single waist sensor was mapped to the pelvis joint of the skeleton, acceleration was converted to m/s^2^ as required, and each window was resampled from 50 Hz to the model’s 20 Hz. Windows were padded to 64 samples, the shortest length that covers a resampled 2.56 s window, rather than to the model’s 200-sample default. The default would have repeated the same window several times over and distorted its temporal content. The frozen and fine-tuned readouts used identical padding so that they remain comparable. An audit of the publicly released checkpoint established that it accepts three input channels only (inferred input channels = 3; first-layer batch-normalisation width = 66 = 22 joints × 3). Its first layer is built to receive three values per skeleton joint, leaving no input position into which a gyroscope channel could be supplied. It is therefore an accelerometer-only model, consistent with the other open motion foundation models, RelCon [18], Inertia-1 [19], and the large-scale sensor model of [20], and with the authors’ own released evaluation configuration. The gyroscope channel is therefore unavailable to the released model, a constraint of the artefact rather than of our usage.

We read out the foundation model in three ways that differ in how much the pretrained model is allowed to change on our data, from using it unchanged to retraining it fully. A frozen linear probe holds the pretrained encoder fixed and fits only a simple linear classifier on its 512-dimensional output. This is the standard way of asking what a pretrained model already represents without allowing it to adapt. The second readout keeps the same frozen output but classifies it with XGBoost, so that the representation, rather than the classifier, distinguishes it from the hand-crafted arms. Full fine-tuning, in contrast, trains the graph-convolutional encoder together with a linear head end-to-end from the released weights, allowing the representation itself to adapt to the task.

### 2.5. Labelling-Requirement Analysis

To estimate how much bedside observation a prospective ward study would have to fund, the six-axis and accelerometer-only three-axis hand-crafted models were retrained on reduced labelled budgets of 5, 10, 20, 40, and 80 min and on the full training set of approximately 156 min. The held-out test subjects were unchanged at every budget. The sampling unit was a whole labelled activity bout rather than an individual window, because a nurse or observer annotates continuous stretches of behaviour rather than disconnected 2.56 s snippets. Sampling windows at random would also have let 50%-overlapping neighbours inflate a small budget. Within each training fold, bouts were drawn in random order until the minute budget was exhausted. Sampling was deliberately left unstratified, so that a small budget may contain no bed-exit event at all, which is the situation a short observation period would actually produce. Each budget was run with three seeds, each reseeding both the bout subsample and the classifier, and the reported values are means over those seeds. For each budget we also recorded how many bed-exit bouts the training data actually contained, so that performance could be read against event count as well as against observation time.

### 2.6. Validation and Statistical Analysis

All models were evaluated with subject-grouped five-fold cross-validation (stratified group *k*-fold), so that no subject appeared in both training and test folds. Within-subject leakage across the train/test boundary inflates wearable-HAR performance and was excluded by construction [30]. The unit of analysis was the subject. For each held-out subject, we computed the macro-averaged F1 over the classes that subject performed. All comparative statistics were paired across subjects (n = 30), not across folds or windows, because fold-level statistics (n = 5) cannot support inference. Three descriptive analyses are nevertheless reported without per-subject inference and are labelled as such throughout: the aggregate score of the twelve-class reference problem and the window-length sweep (both fold-level), and the deployment scoring of Section 3.6 and Section 3.7 (sensitivities and false-alarm rates pooled over windows). None carries a *p*-value or a confidence interval, and every inferential comparison in this paper is per-subject. Macro-F1 was the primary metric, chosen over accuracy because the transition classes are rare and would be masked by the abundant static classes. In plain terms, F1 combines how often a predicted class is correct (precision) with how many of its true cases are caught (recall) on a 0–1 scale. Macro-F1 averages this across all ten classes, so that recognising the common postures well cannot compensate for missing the rare bed-exit transitions. Because catching a patient leaving the bed is the clinically decisive outcome, two further quantities are reported for the two bed-exit transitions. The first is recall (sensitivity), the proportion of true bed-exit windows the model detects. The second, called bed-exit F1 throughout, is the mean per-subject F1 of the two bed-exit classes, that is, how reliably the model flags a patient rising from bed.

The 50% window overlap makes neighbouring windows statistically dependent, which would inflate significance if windows were the unit of inference. They are not. Each subject contributes exactly one number to every comparison (their own macro-F1 or bed-exit F1), and the paired tests, effect sizes, and bootstrap intervals all resample subjects (n = 30), never windows. Overlap-induced correlation is therefore contained inside a single subject’s score, where it affects how precisely that subject’s performance is estimated. It cannot enlarge the effective sample size of the inference or narrow the reported confidence intervals. The same reasoning covers the unequal number of windows per subject, since each subject is weighted equally regardless of how many windows they contributed.

Because every model was evaluated on the same 30 subjects, methods were compared subject by subject rather than in aggregate. Paired comparisons between representations used the Wilcoxon signed-rank test, a non-parametric paired test. The effect size was the matched-pairs rank-biserial correlation, which expresses the net proportion of subjects favouring one condition and reaches 1.0 when every subject does. A percentile bootstrap (a resampling method; 10,000 resamples over subjects) gave the 95% confidence interval (CI) of the mean paired difference. All reported paired differences and their CIs were computed from the unrounded per-subject values and may therefore differ in the final decimal place from the subtraction of the rounded summary means shown. Because several methods were compared against the same reference, these *p*-values were Holm-corrected to guard against false positives from repeated testing. For reproducibility, the neural-network training used standard settings. Fine-tuning minimised class-weighted cross-entropy with the Adam optimiser (learning rate 1 × 10^−4^, batch size 64) for up to 20 epochs. The training epoch was selected on an inner validation split held out by subjects from the training folds, never on the test subjects, to avoid tuning on the test set. Class weighting was used in this neural-network training only, where it prevented the rare transitions from collapsing. The optimiser settings were likewise fixed in advance rather than tuned on these data.

### 2.7. Reproducibility, Software, and Ethics

Analyses were implemented in Python 3.13 with scikit-learn 1.7.2, XGBoost 3.3.0, SciPy 1.16, and PyTorch 2.13 (Compute Unified Device Architecture, CUDA 12.6); fine-tuning ran on a single NVIDIA GeForce RTX 4070 Laptop graphics processing unit (GPU; 8 GB; NVIDIA Corporation, Santa Clara, CA, USA). Random seeds were fixed, and the library versions above were pinned. The dataset is public and fully de-identified, and no new human data were collected; the study was granted an exemption from institutional review board review by the Samsung Medical Center Institutional Review Board (protocol SMC 2026-08-024). Dataset licences and access routes are given in the Data Availability Statement. This is a methodological feasibility benchmark on a public dataset rather than a clinical prediction model development or validation study or a diagnostic accuracy study, so no single clinical reporting checklist (TRIPOD+AI, the artificial intelligence extension of the Transparent Reporting of a multivariable prediction model for Individual Prognosis Or Diagnosis statement; STARD-AI, the corresponding extension of the Standards for Reporting of Diagnostic Accuracy Studies) applies in full. We nonetheless followed their model-reporting and validation-transparency items where relevant, and the planned prospective ward study will be reported in accordance with the Strengthening the Reporting of Observational Studies in Epidemiology (STROBE) statement. All results were reproduced in a clean, isolated Python environment with the pinned library versions: the hand-crafted-feature and gradient-boosted-tree results reproduced exactly, and the foundation-model embeddings were bit-identical. Full fine-tuning, which is not bit-reproducible on the GPU, was run over three seeds (macro-F1 0.774 ± 0.019). The between-seed spread was largest for the rare bed-exit classes.

The computational footprint confirms that the deployable configuration runs on commodity hardware. The six-axis hand-crafted pipeline used no GPU: feature extraction over all 7978 windows took 0.8 s and fitting one 400-tree gradient-boosted model took 9.2 s on a central processing unit (CPU). Full fine-tuning of the foundation model, by contrast, required approximately 8 min per cross-validation fold and 3.1 GB of memory on the 8 GB laptop GPU. The model that a ward would deploy is therefore trainable in seconds without specialised hardware.

## 3. Results

### 3.1. Ward-Relevant Task and the Composition of the Headline Score

The 2.56 s windowing of HAPT yielded 7978 windows for the ten-class ward task across all 30 subjects. On the full twelve-class HAPT problem, the hand-crafted six-axis model reached an accuracy of 0.937 and a fold-level macro-F1 of 0.854. Per-class F1 from the same model ranged from 0.999 for lying and 0.952–0.958 for the walking and stair classes down to 0.691 for lie-to-stand and 0.699 for lie-to-sit (Figure 2). The mean F1 of the two stair classes was 0.956 and that of the six postural transitions was 0.760. On the ten-class ward task, the same model reached a per-subject macro-F1 of 0.822 ± 0.102 and a bed-exit F1 of 0.657 ± 0.255. The lowest per-class values were lie-to-stand (0.662) and sit-to-lie (0.701) (Table 2). Expressed as sensitivity, the six-axis model detected 71% of lie-to-sit and 63% of lie-to-stand transitions (recall 0.71 and 0.63; precision 0.71 and 0.70). Across the 30 subjects, the two bed-exit classes were represented by 169 test windows in total (85 lie-to-sit and 84 lie-to-stand). Because evaluation used subject-grouped cross-validation, each window served once as a held-out test case, so these counts bound the precision of the bed-exit metrics. In the ten-class confusion matrix (Figure 3; a table showing what each activity was mistaken for), 302 of the 312 static-class misclassifications were confusions between sitting and standing. The directional transitions were most often confused with the transition sharing their lying endpoint rather than with their direction-reversed counterpart.

### 3.2. Contribution of the Gyroscope

Removing the gyroscope and retaining the three accelerometer axes reduced per-subject macro-F1 from 0.822 to 0.647, a paired difference of 0.176 (95% CI 0.144–0.208; Wilcoxon *p* = 5.6 × 10^−9^; rank-biserial 0.99; 29 of 30 subjects favoured the six-axis configuration). This and all reported differences are computed on the unrounded per-subject values and may therefore differ by 0.001 from the difference in the rounded figures shown, as noted in Section 2.6. Bed-exit F1 fell from 0.657 to 0.351, a difference of 0.306 (95% CI 0.203–0.416; *p* = 3.4 × 10^−5^; 26 of 30 subjects). The per-class gains from the gyroscope were 0.002 for lying and 0.001 for walking, 0.088 and 0.083 for sitting and standing, and, for the transitions, 0.369 for sit-to-stand, 0.310 for lie-to-stand, 0.275 for lie-to-sit, and 0.253 for sit-to-lie (Figure 4). The per-subject values behind these paired tests are shown in Figure 5.

The mirror ablation, retaining the three gyroscope axes and removing the accelerometer, separates the two contributions instead of only removing one sensor. The gyroscope-only model reached a per-subject macro-F1 of 0.706 and a bed-exit F1 of 0.512. It exceeded the accelerometer-only model on both (macro-F1 +0.059, 95% CI 0.015–0.106, *p* = 0.011, 21 of 30 subjects; bed-exit F1 +0.161, 95% CI 0.047–0.275, *p* = 0.011, 19 of 30 subjects). It remained below the six-axis model (macro-F1 lower by 0.117, 95% CI 0.084–0.148, *p* = 1.7 × 10^−6^, 27 of 30 subjects). The two three-axis arms divide the task between them. The accelerometer was far better for lying (0.996 against 0.696) and slightly better for sitting and standing, whereas the gyroscope was better for every one of the six directional transitions, by 0.119 to 0.253 (Table 2). Gravity therefore carries the static posture and rotation carries the transition, which is why neither three-axis configuration approaches the six-axis model.

### 3.3. Window Length

On the common set of activity bouts long enough to be sampled at every window length, fold-level macro-F1 rose with window length from 0.720 at 0.64 s to 0.764 at 1.28 s, 0.806 at 1.92 s, and 0.833 at 2.56 s. The mean transition F1 rose over the same range from 0.589 to 0.761, and bed-exit F1 from 0.557 to 0.686. At the 2.56 s length, 40 of the labelled bouts were shorter than one window and were not sampled: 29 sit-to-stand, 7 stand-to-sit, and 4 bed-exit bouts (2 lie-to-sit and 2 lie-to-stand). The exclusion therefore fell almost entirely on the sit-to-stand and stand-to-sit classes, which are not bed-exit targets, and removed 4 of the 118 bed-exit bouts in the corpus. The sweep is tabulated with the corresponding window counts in Table 3 and plotted in Figure S3. Shortening the window from 2.56 s to 0.64 s raised the number of windows from 7978 to 35,473 while lowering every score.

### 3.4. Label Efficiency

With the six-axis hand-crafted model, per-subject macro-F1 (mean over three seeds, each reseeding both the label subsample and the classifier) increased with the labelled training budget from 0.435 at 5 min to 0.737 at 40 min and 0.785 at 80 min. It reached 0.815 at the full budget of approximately 156 min, so the value at 80 min was 96% of the full-budget value (Figure 6a). Bed-exit F1 over the same budgets rose from 0.180 at 5 min to 0.492 at 40 min and 0.557 at 80 min and continued to 0.640 at the full budget and did not plateau (Figure 6b). The full-budget values here are three-seed means and differ for that reason alone from the single-run values of 0.822 and 0.657 reported in Section 3.5. The mean number of bed-exit bouts present in the training data, over the same three seeds, was 2.7 at the 5 min budget, 25.1 at 40 min, and 91.2 at the full budget. The accelerometer-only three-axis model remained below the six-axis model at every budget.

### 3.5. Representation Comparison at Full Labels

At full labels, per-subject macro-F1 was 0.822 for the six-axis hand-crafted model, 0.774 for the fully fine-tuned foundation model, 0.731 for its frozen linear probe, 0.706 for the gyroscope-only hand-crafted model, 0.701 for its embeddings with XGBoost, and 0.647 for the accelerometer-only hand-crafted model (Table 4). The fine-tuned model, whose GPU training is not bit-reproducible, is reported throughout as a mean over three seeds (macro-F1 0.774 ± 0.019, bed-exit F1 0.610 ± 0.021); the other arms are deterministic single runs. Bed-exit F1 followed the same ordering, from 0.657 for the six-axis model to 0.351 for the accelerometer-only model.

Against the accelerometer-only hand-crafted baseline, which takes the same input as the foundation model, the frozen linear probe was higher by 0.084 (95% CI 0.048–0.122; *p* = 1.9 × 10^−4^; rank-biserial 0.74). Full fine-tuning was higher in every seed, by a mean of 0.127 (per-seed range 0.112–0.149; paired *p* ≤ 6 × 10^−6^). The pretraining was clearly not inert. Against the six-axis hand-crafted model, however, full fine-tuning was lower in all three seeds, by a mean of 0.049 in macro-F1 (per-seed range 0.027–0.064; paired *p* = 4 × 10^−4^, 3 × 10^−3^ and 5 × 10^−2^). The six-axis advantage was thus consistent in direction across seeds and significant in two of the three. Bed-exit F1 was likewise higher for the six-axis model in every seed, though not significantly (per-seed *p* = 0.33–0.68). Representation contrasts against a common reference were Holm-corrected across the family. The six-axis hand-crafted model at a 40 min labelling budget (macro-F1 0.737) exceeded the accelerometer-only frozen probe and embedding readouts evaluated at the full labelling budget (0.731 and 0.701). Three gyroscope channels with 75 hand-crafted features likewise reached a macro-F1 of 0.706, comparable to the embedding readout of the pretrained accelerometer-only model (0.701). This is a descriptive comparison between arms, not a paired test.

The ordering of representations was not an artefact of the chosen classifier. Repeating the six-axis against accelerometer-only comparison under the same subject-grouped folds with a random forest and with a scaled multinomial logistic regression preserved both the direction and the significance of the difference. The random forest gave +0.118 (95% CI 0.089–0.147, *p* = 8.0 × 10^−8^, 29 of 30 subjects) and the logistic regression +0.111 (95% CI 0.080–0.145, *p* = 3.5 × 10^−8^, 28 of 30 subjects), against +0.176 for the gradient-boosted trees used throughout. The gradient-boosted setting was not the one most favourable to the six-axis input in absolute terms either: the accelerometer-only model scored higher under the random forest and the logistic regression (0.671 and 0.685) than under gradient-boosted trees (0.647).

### 3.6. From Class Scores to an Alarm: Thresholds and Calibration

A ward alarm fires on a predicted probability rather than on the arg-max class, and how well those probabilities are calibrated determines whether any threshold placed on them means what it appears to mean [31]. We therefore scored the binary question of whether a window is a bed exit as the summed probability of the two bed-exit classes from the six-axis model, pooling the out-of-fold predictions of all 7978 windows. Discrimination was high (area under the receiver operating characteristic (ROC) curve 0.998; area under the precision–recall curve 0.971 at a prevalence of 0.021). The probabilities were close to calibrated: the Brier score was 0.0021 against 0.0207 for an uninformative predictor, and the mean predicted probability (0.0201) matched the observed rate (0.0212). The reliability curve was well behaved at both ends (0.0005 predicted against 0.0009 observed in the lowest decile; 0.985 against 0.993 in the highest) but sparsely populated in between, because the model concentrates its probabilities near 0 and 1.

Sweeping the alarm threshold traded missed events against false alarms. At a threshold of 0.20 the model reached a sensitivity of 0.941 with 4.0 window-level false alarms per hour; at 0.35, 0.929 with 2.9 per hour; at 0.50, 0.899 with 2.2 per hour; and at 0.70, 0.870 with 0.7 per hour. False alarms per hour is reported because, unlike precision, it does not depend on the prevalence of bed exits in the test data. The rates are nevertheless specific to this corpus: the scripted recordings contain none of the ward activities, such as being turned, repositioned, or assisted, that would generate false alarms in practice, so they do not predict the false-alarm rate of a device on a hospital ward. The quantity is still the right one to report, because it governs whether such a device would be tolerated: alarms that are frequent and mostly not actionable desensitise staff to the ones that matter, a patient-safety problem in its own right [32].

This binary framing also explains the per-class recall reported in Section 3.1. The model assigned 22% of lie-to-sit windows and 26% of lie-to-stand windows to the *other* bed-exit class rather than to a non-bed-exit class. Recall for the specific upright endpoint was therefore 0.71 and 0.63, while recall for leaving the bed at all was 0.93 and 0.89 (0.91 pooled over the 169 bed-exit windows).

### 3.7. Event-Level Detection and the Cost of Boundary-Crossing Windows

Frame-level and event-level scores answer different questions in continuous recognition, and can diverge whenever events are fragmented, merged, or shifted relative to their annotation [33]. Because a bed-exit bout yields only about 1.5 windows, window-level scores and event-level detection can differ here too. Of the 118 bed-exit bouts in the corpus, 114 produced at least one window. Counting a bout as detected when at least one of its windows was flagged, event-level sensitivity at a threshold of 0.20 was 0.965 over those 114 bouts, slightly above the window-level sensitivity of 0.941. A majority-vote rule gave 0.947. Window-level scoring therefore did not overstate event detection here. It does, however, fall to the window-level figure once the four bouts that were too short to yield any window are counted as missed, which lowers event-level sensitivity to 0.932. Merging consecutive flagged windows into single alarms, as a device would, reduced the false-alarm rate from 4.0 to 3.2 events per hour at a threshold of 0.20, and from 2.2 to 1.4 at the arg-max operating point.

The main analysis places every window strictly inside one annotated bout, which a sliding window in a ward cannot do. Re-scoring the held-out subjects on windows slid continuously across the 642 contiguous stretches of the recordings, so that windows straddle the moment a transition begins, showed what that costs. Because HAPT leaves unannotated gaps between many bouts, these stretches contain 111 of the 169 interior bed-exit windows, which is why the interior scores below differ slightly from the full-set values in Table 4. Most bed-exit windows in such a stream are boundary-crossing (176 of 287). A bed-exit transition lasts only two to three seconds, so a 2.56 s window placed on it usually also covers the adjacent lying or sitting segment. Performance on the boundary-crossing windows was substantially lower than on the interior ones (macro-F1 0.627 against 0.823; bed-exit F1 0.474 against 0.686). Over the combined stream, macro-F1 therefore fell from 0.823 to 0.754 and bed-exit F1 from 0.686 to 0.578, and pooled bed-exit sensitivity fell from 0.937 on the interior windows to 0.833. Labelling each window by its centre sample or by its majority class gave the same result (macro-F1 0.7537 and 0.7538). HAPT annotates only its activity bouts and leaves gaps of 0.1 to 54 s between many of them, so a fully continuous recording cannot be scored without inventing labels for those gaps. The analysis is therefore confined to the boundary effect, and the residual question is left to the prospective ward study.

## 4. Discussion

In clinical terms, the movements that matter most for fall prevention, the moments a patient gets out of bed, were also the hardest for a body-worn sensor to recognise. Ordinary ward activities such as lying still and walking were recognised well. The difficulty, however, lies in a specific place. Naming the exact upright endpoint a patient reaches was unreliable (recall 0.71 for lie-to-sit and 0.63 for lie-to-stand). Most of those errors, however, were confusions between the two bed-exit transitions rather than missed departures. Recall for leaving the bed at all was 0.93 and 0.89, and as a binary alarm the same model flagged 93% of bed-exit windows at roughly three false alarms per hour in these recordings. What a prospective study would work to raise before clinical use is therefore the resolution of the endpoint, and the robustness of the alarm on a continuous stream, rather than the detection of departure as such. Recognising these bed-exit moments depended on capturing the rotation of the body, which the gyroscope provides and which a larger pretrained model could not supply on its own. For a ward considering wearable monitoring, the practical lesson from the configurations tested is that including the gyroscope did more than adopting a larger pretrained model that lacks it.

On a public six-axis inertial dataset, the basic activities were well recognised, near-ceiling for lying and walking, with sitting and standing the main confusion. The postural transitions were the weakest classes, the single weakest being a bed-exit transition. The information that separated these transitions resided almost entirely in the gyroscope. The labelled data required to learn them was governed by how many transition events were observed rather than by total recording time. A pretrained motion foundation model, although it clearly benefited from large-scale pretraining, did not match a simple six-axis hand-crafted model. Together, these findings speak to a single practical point, limited to the configurations compared here: the choice of what to sense mattered more than the scale of the model that interpreted it. Because the six-axis hand-crafted model and the accelerometer-only foundation model differ in both input and model family, the comparison cannot separate scale from input, and it does not show that scale would be unimportant if the inputs were matched.

The comparison across representations makes this concrete, but the argument is structural before it is empirical. An audit of the released weights shows that no accelerometer-only motion foundation model, whatever its scale, has an input position for the gyroscope, so the conclusion does not hinge on which single model was benchmarked. Full fine-tuning lifted the accelerometer-only foundation model well above an accelerometer-only hand-crafted baseline built on the same input, so the pretraining was not inert. Yet the same fine-tuned model remained below a six-axis hand-crafted model that trains in seconds on a laptop, and the six-axis model reached that advantage from a fraction of the labelled data. The gap is not attributable to a weak model but to the input available to it. The publicly released weights of UniMTS accept three accelerometer channels, and the other openly released motion foundation models (RelCon [18], Inertia-1 [19], and the large-scale sensor model of [20]) are likewise accelerometer-centric, reflecting the accelerometer-only composition of the large wearable corpora on which they are trained. Adaptation cannot recover information that the input never carried. We benchmarked one foundation model empirically, so the performance comparison is, strictly, a statement about the released accelerometer-only UniMTS. The broader claim rests on the channel audit rather than on having tested every model, and that audit is not specific to UniMTS. This result is consistent with reports that specialised foundation models often fail to surpass well-tuned supervised baselines outside vision and language [34], and it identifies the mechanism in this domain as a sensor-modality mismatch rather than a modelling deficiency.

These findings refine, rather than repeat, earlier wearable bed-exit work. Prior systems established that a body-worn device can flag a bed exit: a dedicated inertial node on a geriatric ward [11], a battery-less RFID tag validated in hospitalised patients [12], and a gyroscope-bearing inertial measurement unit (IMU) that recognises the lying-to-sitting transition [13]. None, however, isolated which sensor axis carries the transition, benchmarked a pretrained foundation model, or characterised the labelling requirement on reproducible public data. Notably, the last system already used an inertial unit that included a gyroscope yet did not quantify its contribution. Our ablation supplies that missing step, showing that removing the gyroscope roughly halves bed-exit F1. Conversely, the RFID study evaluated real hospitalised patients, which our public-data design does not, reinforcing that our results are an upper bound intended to specify, not replace, such a patient study.

A parallel literature monitors the patient in bed through modalities other than inertial sensing. In-ear acoustic sensing recognises body movement, respiration, and heartbeat from a sleep earbud and maps them onto sleep stages [35], and smartphone systems combine movement with ambient sound for fine-grained sleep-stage tracking [36]. These address a different clinical question from ours, characterising sleep architecture across a night rather than detecting the discrete moment a patient leaves the bed. Neither speaks to which inertial channels carry a directional postural transition, so they are complementary rather than competing approaches. They are instructive here in one respect: each is bounded by what its modality can sense, an acoustic channel resolving respiration and movement but not the direction of a postural change. That is the same constraint we identify for accelerometer-only inertial models, and it suggests the sensor-modality question raised here recurs across unobtrusive in-bed monitoring rather than being specific to the gyroscope.

What this task can and cannot stand for should be stated plainly. A bed exit in a ward is a sequence rather than a single event. A patient shifts to the edge of the mattress, moves the legs off the bed, pushes up, and only then stands or walks away, and a monitor could in principle intervene at any of those stages. HAPT annotates none of that structure. It labels a single directional transition from lying to sitting or to standing, which corresponds to one stage of that sequence, the change in whole-body posture that ends the recumbent state. The transitions studied here are therefore bed-exit-relevant, the term used in the title, and not bed exits in the full clinical sense. The results bear on whether an inertial sensor can recognise the postural change, not on how early in the sequence a device could act. Resolving the earlier stages, and with them the warning time a system could offer, requires bedside-annotated data and is part of the prospective ward study.

The failure mode has a specific clinical shape. Errors concentrated in the distinction between sitting and standing, and this same confusion propagated into the transitions that share a seated or standing endpoint, so that the model more often confused a transition’s upright endpoint than its direction. For a bed-exit monitor, this is a consequential error, because the upright endpoint a patient reaches determines what response is warranted. Resolving that endpoint improved with the gyroscope (sitting and standing each gained about 0.08 in F1), a signal the accelerometer-only models cannot see. Recognising the direction of a bed exit was comparatively reliable; resolving its upright endpoint was not.

The label-efficiency analysis reframes the cost of building such a monitor. Annotated data is the recognised bottleneck in wearable activity recognition. Labelling requires an observer at the bedside rather than only sensor time [25], and scarce labels together with skewed class distributions are repeatedly identified as the field’s central obstacles [15]. That scarcity is precisely what motivates the large self-supervised corpora now used to pretrain wearable models [22]. Overall recognition saturated after a modest amount of labelled observation, but bed-exit performance kept improving up to the largest budget. Because the number of captured bed-exit events grew with observation time, the two are confounded in this design. Nonetheless, the continued improvement of this rare class while the common classes saturated is consistent with an event-count rather than a duration bottleneck. Because bed exits are brief and infrequent, an observation protocol planned in hours will under-sample them: the rare, safety-critical transitions, rather than observation time alone, appear to dominate the labelling requirement.

These conclusions are bounded by the study’s design as a public-data feasibility benchmark, a scope chosen deliberately to settle sensor-configuration questions before a prospective cohort is committed. First, the transition-recognition results derive from a single public cohort of 30 healthy volunteers aged 19–48 performing scripted activities, with no external inertial validation. The patients for whom a bed-exit monitor is built differ from that cohort in ways that bear directly on the signal. They are older and frequently frail. They rise more slowly and in segmented movements rather than one continuous sweep; they push off with the arms or a bed rail, and they may be tethered to lines or drains. Some have the neurological or musculoskeletal impairment that made them a fall risk in the first place. Each of these changes the amplitude and the shape of the rotation the gyroscope sees, and slower transitions also occupy more analysis windows than the two to three seconds observed here. That this degradation is real rather than a cautious assumption is shown by work detecting postural transitions from a single waist-worn inertial sensor in both healthy and patient populations [37]. There, positive predictive value and sensitivity fell from 98% and 95% in healthy participants to 89% and 89% in participants with disease. The public-data results are therefore best read as an upper bound, and the size of the resulting domain gap is itself the reason a prospective ward study with external validation is needed. Having established technical feasibility here, that study can proceed as a STROBE-reported observational cohort on prospectively collected patient data. Second, the clinically relevant contrast is between remaining in bed and leaving it, but the public corpus labels lying only as a static posture, so movements made while a patient stays recumbent are not represented. What was evaluated here is the discrimination of that static state and the transitions returning to it from the transitions leaving it. Whether an inertial monitor can separate in-bed repositioning from the onset of a bed exit, therefore, remains open and requires bedside-annotated data. Third, the foundation model was applied to a single waist sensor mapped to one skeleton joint, with the signal resampled to the model’s rate and padded to cover the analysis window. These choices, made to match the released model, are conservative and may understate its achievable performance. Fourth, the model comparison used frozen and fully fine-tuned readouts of one foundation model with limited architecture search. Full fine-tuning was repeated over three seeds, and although its macro-F1 was stable (0.774 ± 0.019), the six-axis advantage in macro-F1 reached significance in two of the three seeds. That particular margin should be read as consistent in direction rather than uniformly significant. The empirical comparison is accordingly a statement about the released UniMTS checkpoint and not about motion foundation models as a class: no claim is made that a larger or differently trained model could not close the margin on accelerometer input. What does generalise beyond UniMTS is the narrower structural point established by the channel audit: an accelerometer-only model has no input position for the gyroscope, so the rotation signal that carried the transitions here is unavailable to it at any scale. Testing whether a second, independently trained motion foundation model behaves the same way remains future work.

Fifth, the evaluation windows sit inside annotated bouts, whereas a deployed sliding window straddles the moment a transition begins. Re-scoring the held-out subjects across the contiguous stretches of the recordings showed this is not a minor detail. Most bed-exit windows in such a stream cross a boundary, and on those stretches bed-exit sensitivity fell from 0.937 to 0.833 and bed-exit F1 from 0.686 to 0.578. The headline figures should be read with that reduction in mind. Because HAPT leaves long unannotated gaps between its bouts, even this is not a full continuous-monitoring estimate. What a device would achieve over an uninterrupted ward shift remains untested here. Finally, although the alarm probabilities proved close to calibrated on this cohort, calibration was assessed in aggregate and on scripted data from healthy volunteers. The reliability curve is sparsely populated between its extremes, so the mid-confidence range is poorly characterised, and any deployed threshold would have to be recalibrated on ward data. Nor could we stratify performance by demographic or clinical subgroup. The public dataset carries no labels for mobility, assistive-device use, or diagnosis, and only coarse age information. These are precisely the variables that distinguish the patients most at risk of falling, and the ones on which a fairness analysis would have to rest. The prospectively characterised cohort of the planned ward study is designed to record them and to pre-specify those subgroup analyses.

## 5. Conclusions

For recognising the postural transitions that begin a bed exit, on public recordings of healthy volunteers, the sensor configuration mattered more than the scale of the model among the configurations tested: a six-axis hand-crafted model, its accelerometer-only and gyroscope-only ablations, and one accelerometer-only foundation model. The gyroscope carried the signal that distinguishes these transitions: removing it roughly halved bed-exit F1, and the mirror ablation showed that gravity carries the static posture while rotation carries every one of the six directional transitions. A six-axis hand-crafted model outperformed a pretrained accelerometer-only motion foundation model even after full fine-tuning and with far less labelled data. That empirical comparison concerns one released model, and because the six-axis and foundation-model arms differ in both sensor input and model family, it does not separate model scale from input. What generalises beyond it is the structural point that an accelerometer-only model has no input position for the gyroscope, so the rotation signal is unavailable to it at any scale. The labelling requirement appeared to scale with the number of rare transition events rather than with observation time alone.

These are upper-bound estimates. Scored on windows that cross bout boundaries, as a deployed sliding window must, bed-exit sensitivity fell from 0.937 to 0.833 on the stretches scored, and the cohort is healthy volunteers performing scripted activities rather than ward patients. The study therefore shows that a waist-worn sensor can recognise the postural transitions that begin a bed exit; it does not demonstrate bed-exit detection in a hospital ward, and no claim of clinical performance follows from it. Within those bounds, the findings specify concrete inputs for the planned prospective ward study: a sensor package that includes the gyroscope, and a labelling protocol sized by target bed-exit event count rather than by observation hours.

## Figures and Tables

**Figure 1:**
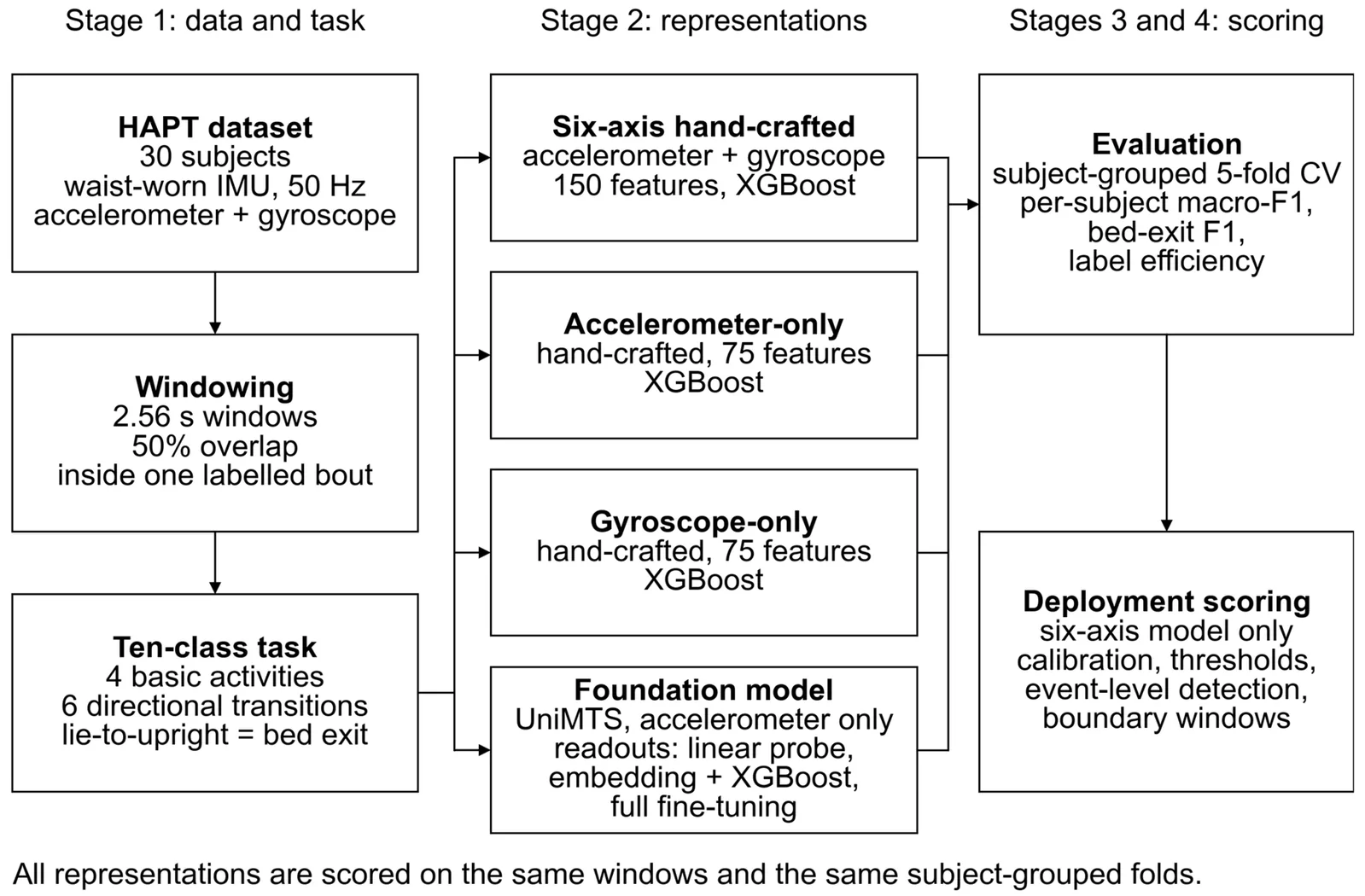
Study pipeline in four stages: the public HAPT recordings, windowing, and the ten-class task (Stage 1); the four representations compared on the same windows and folds (Stage 2); the subject-grouped evaluation (Stage 3); and the deployment scoring of the six-axis model (Stage 4). Section 2.1 describes each stage. HAPT, Human Activities and Postural Transitions dataset; IMU, inertial measurement unit; CV, cross-validation.

**Figure 2:**
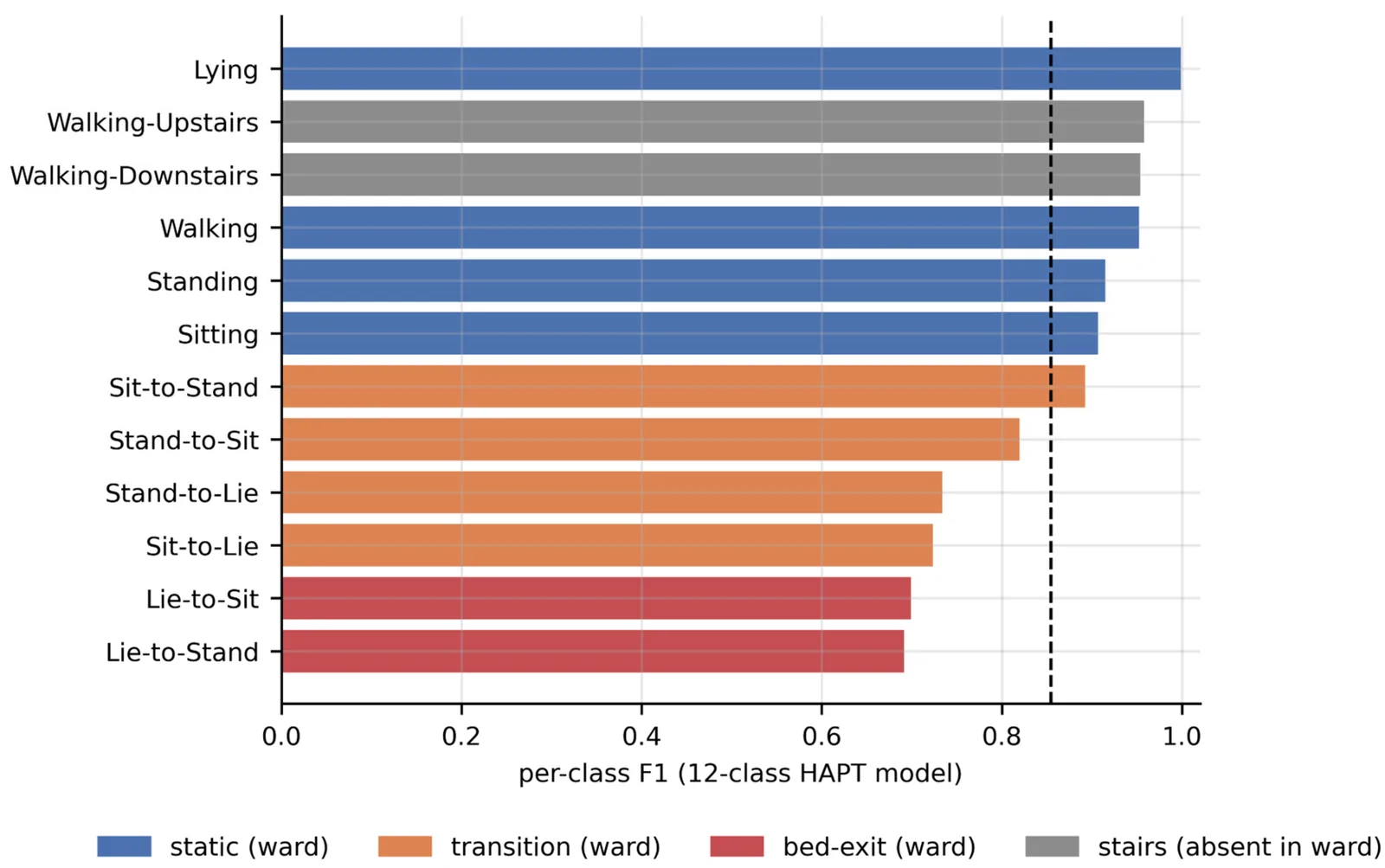
Per-class F1 of the standard 12-class HAPT model. The headline macro-F1 is carried by stairs, which are absent in a ward, and by static postures, whereas the two bed-exit transitions are the weakest classes. The dashed line marks the fold-level macro-F1 of the twelve-class model (0.854).

**Figure 3:**
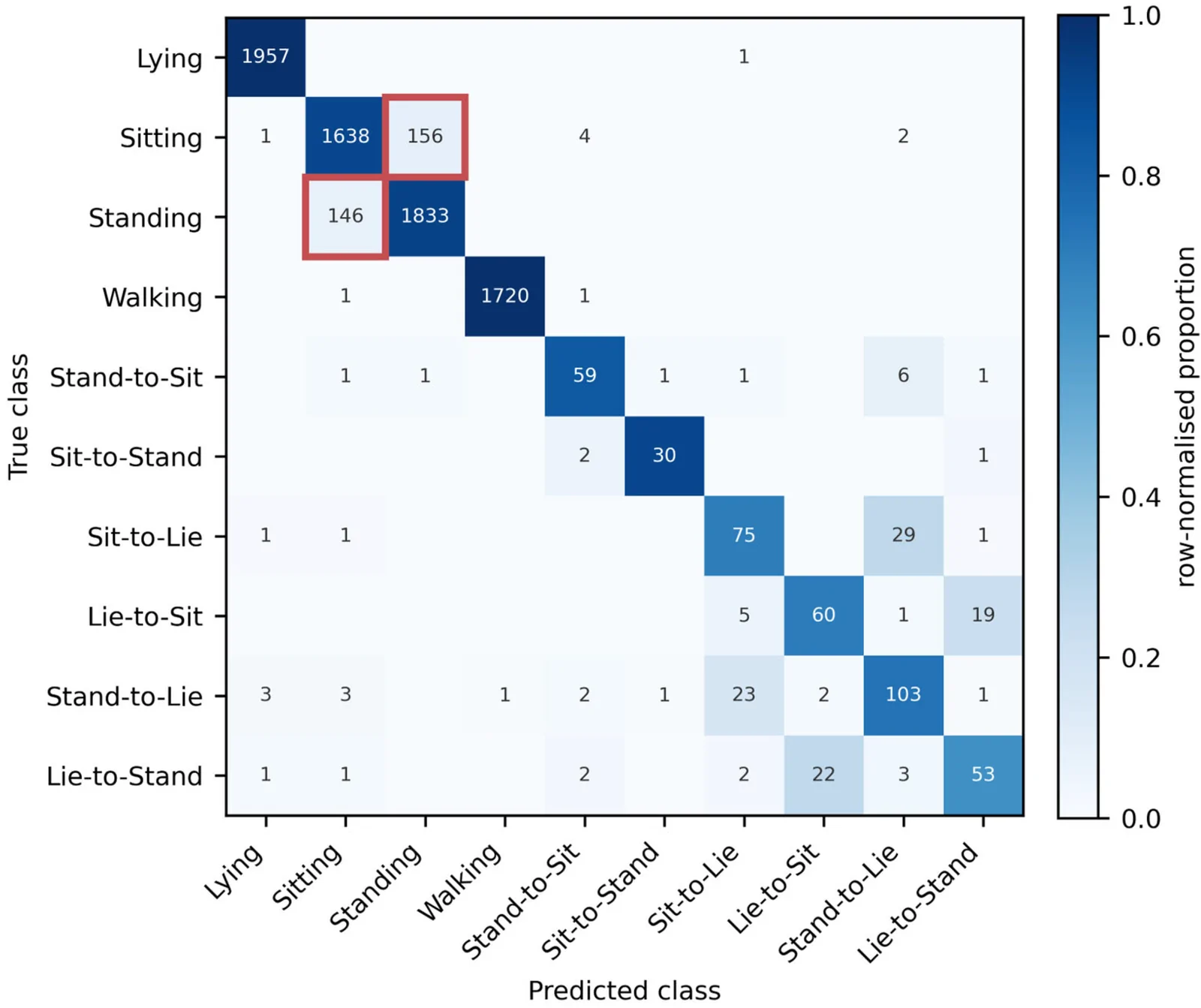
Row-normalised confusion matrix of the ten-class ward task (2.56 s windows, six-axis hand-crafted model). Most static-class errors are sitting–standing confusions (outlined); directional transitions are most often confused with the transition sharing their lying endpoint.

**Figure 4:**
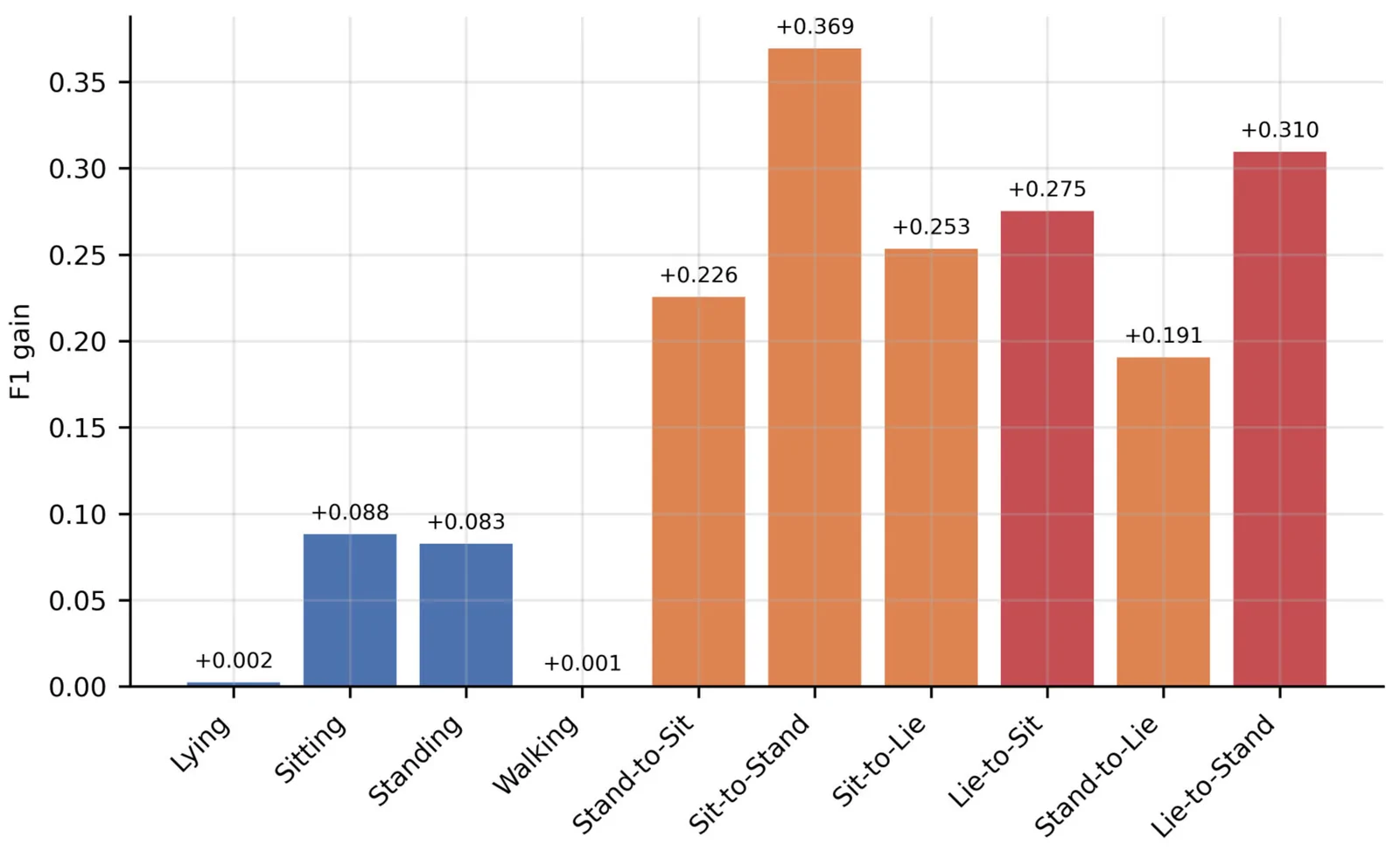
Per-class F1 gain from adding the gyroscope (six-axis minus accelerometer-only, same pipeline). Lying and walking are unaffected and sitting and standing gain about 0.08, whereas every postural transition, and the bed-exit transitions in particular, gain 0.19 to 0.37.

**Figure 5:**
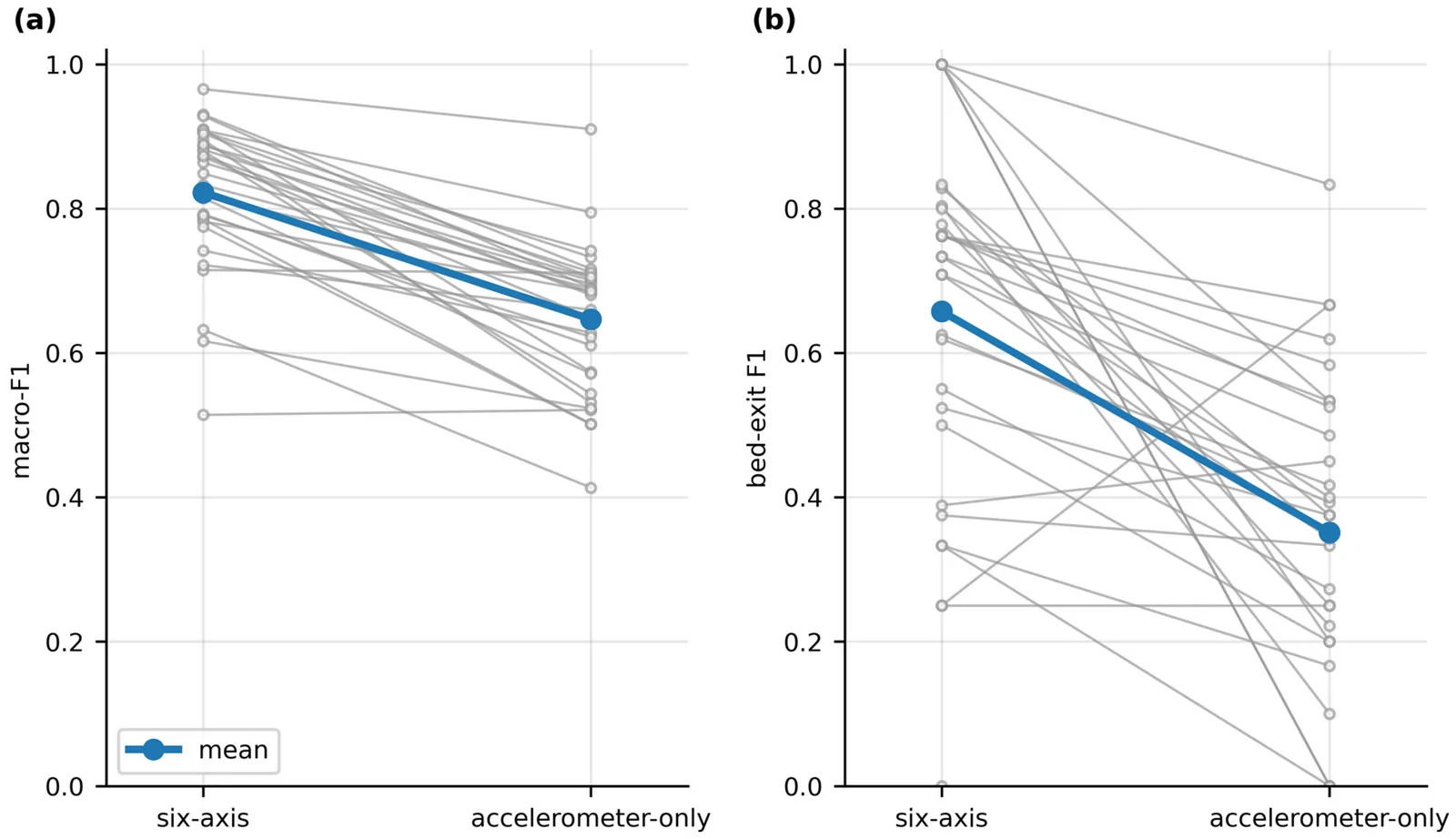
Per-subject scores with and without the gyroscope. Each grey line is one of the 30 subjects; the blue line is the mean. (**a**) Macro-F1; (**b**) bed-exit F1. The paired structure shown here is the unit on which every statistical comparison in this study is made.

**Figure 6:**
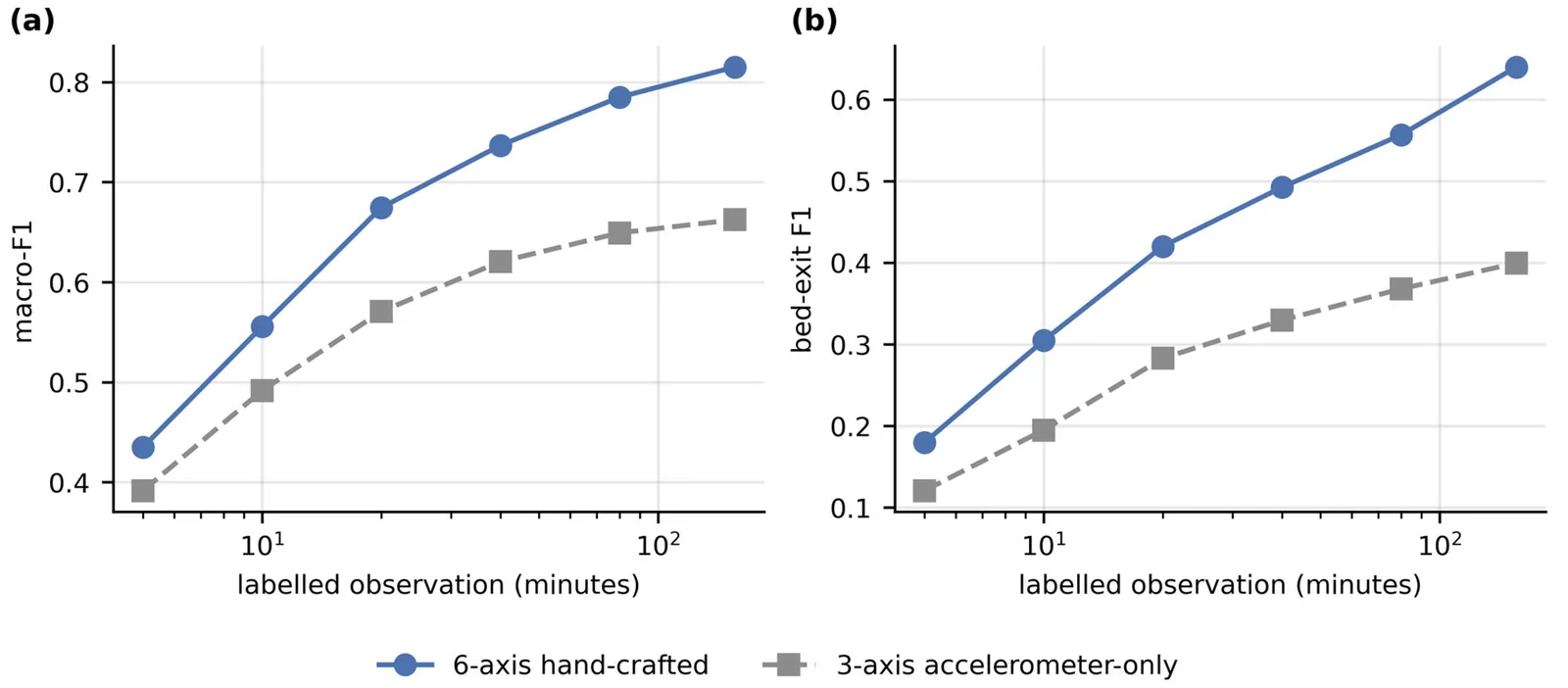
Label-efficiency curves for the deployable hand-crafted models against labelled observation time. (**a**) Macro-F1; (**b**) bed-exit F1.

**Table 1:** Acquisition device and sensor specifications of the HAPT dataset. Full-scale ranges are those reported by the device sensors; the dataset itself does not specify a measurement range.

Component	Specification
Device	Samsung Galaxy S II (Samsung Electronics, Suwon, Republic of Korea), waist-mounted
Accelerometer	STMicroelectronics (Geneva, Switzerland) K3DH, triaxial, full-scale range ±2 g
Gyroscope	STMicroelectronics K3G, triaxial, ±500°/s
Sampling rate	50 Hz (accelerometer and gyroscope)

**Table 2:** Per-class F1 across representations at full labels (subject-grouped cross-validation, 2.56 s windows). Values are pooled per-class F1 computed over all windows; the bed-exit F1 reported in the text is instead the mean of the two bed-exit classes’ per-subject F1 and therefore differs slightly from the average of the corresponding cells here.

Class	Hand-Crafted 6-axis + XGBoost	Hand-Crafted 3-axis Accelerometer-Only + XGBoost	Hand-Crafted 3-axis Gyroscope-Only + XGBoost	UniMTS Embedding + XGBoost	UniMTS Frozen Linear Probe	UniMTS Full Fine-Tune
Lying	0.998	0.996	0.696	0.967	0.979	0.992
Sitting	0.912	0.824	0.758	0.814	0.822	0.825
Standing	0.924	0.841	0.820	0.841	0.847	0.834
Walking	0.999	0.998	0.996	0.999	0.999	0.998
Stand-to-Sit	0.843	0.617	0.746	0.696	0.726	0.830
Sit-to-Stand	0.923	0.554	0.806	0.831	0.831	0.907
Sit-to-Lie	0.701	0.448	0.648	0.527	0.535	0.619
Lie-to-Sit	0.710	0.435	0.554	0.457	0.541	0.642
Stand-to-Lie	0.728	0.537	0.700	0.560	0.602	0.618
Lie-to-Stand	0.662	0.353	0.548	0.431	0.521	0.617

**Table 3:** Recognition against analysis-window length on the survivorship-controlled segment set. Shorter windows yield more windows but less information per window; the transition classes lose the most. Values are fold-level and descriptive.

Window Length (s)	Windows	Macro-F1	Macro-F1 SD	Mean Transition F1	Bed-Exit F1
0.64	35,473	0.720	0.028	0.589	0.557
1.28	17,146	0.764	0.008	0.643	0.610
1.92	11,024	0.806	0.018	0.715	0.680
2.56	7978	0.833	0.052	0.761	0.686

**Table 4:** Headline metrics (per-subject macro-F1 and bed-exit F1) by representation, ordered by macro-F1.

Representation	Input	Macro-F1	Bed-Exit F1
Hand-crafted 6-axis + XGBoost	6-axis (accel + gyro)	0.822	0.657
UniMTS full fine-tune	3-axis (accelerometer)	0.774	0.610
UniMTS frozen linear probe	3-axis (accelerometer)	0.731	0.534
Hand-crafted 3-axis gyroscope-only + XGBoost	3-axis (gyroscope)	0.706	0.512
UniMTS embedding + XGBoost	3-axis (accelerometer)	0.701	0.443
Hand-crafted 3-axis accelerometer-only + XGBoost	3-axis (accelerometer)	0.647	0.351

## Data Availability

The data presented in this study are available in the UCI Machine Learning Repository at https://doi.org/10.24432/C54G7M (dataset 341; accessed on 22 September 2026). These data were derived from the following resource available in the public domain: the Smartphone-Based Recognition of Human Activities and Postural Transitions (HAPT) dataset [23]. The analysis code and a reproduction notebook are available at https://github.com/youmipark329-smc/spg-imu-bed-exit (accessed on 22 September 2026) under the MIT licence; the UniMTS foundation model and its checkpoint are obtained separately from the original authors and are not redistributed. The reported classical and foundation-model results were reproduced in a clean, version-pinned environment.

## Supplementary Materials

The following supporting information can be downloaded at: https://www.mdpi.com/article/10.3390/s26196073/s1, Table S1: The twelve HAPT classes and their inclusion in the ten-class ward-relevant task; Figure S1: Full-label ward-task performance of every representation, plotted as macro-F1 against bed-exit F1; Figure S2: Raw six-axis signals as supplied by HAPT, with no filtering, detrending, or per-recording normalisation, for three static postures and one bed-exit transition; Figure S3: Recognition against analysis-window length, computed on the segments long enough to yield a window at every length so that the comparison is not confounded by survivorship.

## Abbreviations

The following abbreviations are used in this manuscript:

HAR	human activity recognition
HAPT	Human Activities and Postural Transitions dataset
IMU	inertial measurement unit
