# Supplementary material for: Sensor Modality Versus Pretraining for Recognising Bed-Exit–Relevant Postural Transitions: A Gyroscope-Focused Feasibility Study on Public Wearable-IMU Data
Source: Sensors (Basel). 2026 Sep 25;26(19):6073. doi: 10.3390/s26196073 (PMC13667495; doi:10.3390/s26196073)
Supplement: Supplementary file 1 [file sensors-26-06073-s001.zip › sensors-4514821-supplementary.pdf]

sensors
MDPI

Supplementary Materials

Supplementary Materials: Sensor modality versus pretraining for recognising bed-exit–relevant postural transitions: a gyroscope-focused feasibility study on public wearable-IMU data

Table S1. The twelve HAPT classes and their inclusion in the ten-class ward-relevant task. Stair ascent and descent were excluded because they do not occur in a typical ward, are easily separated (inflating aggregate accuracy), and are unrelated to the postural-transition target.

ID	Class	Type	Ward task	Reason
1	WALKING	Dynamic (locomotion)	Included	Common ward locomotion
2	WALKING_UPSTAIRS	Dynamic (locomotion)	Excluded	Absent in a ward; easily separated (inflates accuracy); unrelated to the target
3	WALKING_DOWNSTAIRS	Dynamic (locomotion)	Excluded	Absent in a ward; easily separated (inflates accuracy); unrelated to the target
4	SITTING	Static (posture)	Included	Basic ward posture
5	STANDING	Static (posture)	Included	Basic ward posture
6	LAYING	Static (posture)	Included	Proxy for remaining on the bed
7	STAND_TO_SIT	Transition	Included	Postural transition (sitting down)
8	SIT_TO_STAND	Transition	Included	Postural transition (standing up)
9	SIT_TO_LIE	Transition	Included	Return-to-bed transition
10	LIE_TO_SIT	Transition	Included	Bed-exit (fall precursor; primary target)
11	STAND_TO_LIE	Transition	Included	Return-to-bed transition
12	LIE_TO_STAND	Transition	Included	Bed-exit (fall precursor; primary target)

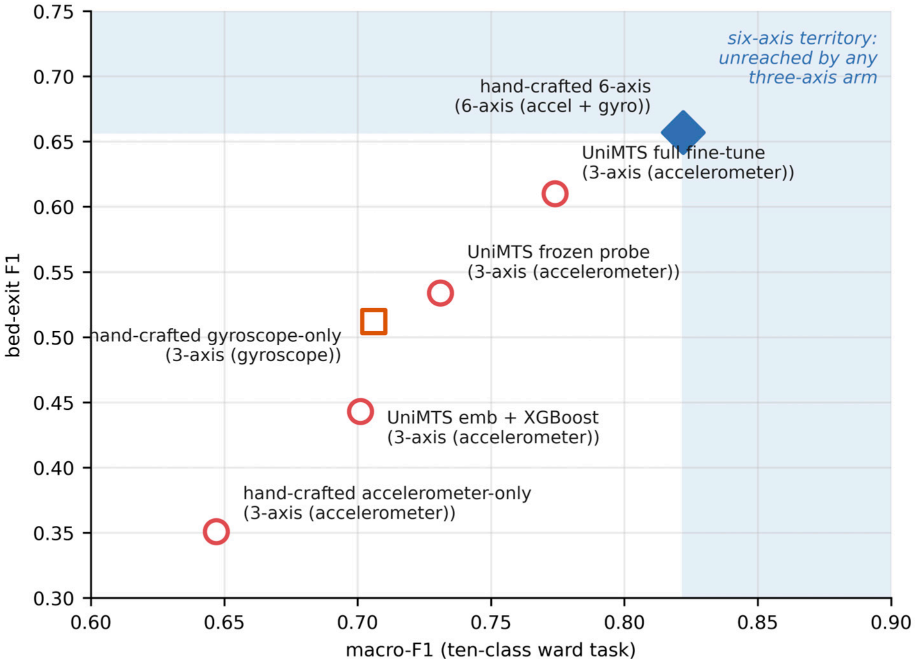

Figure S1. Full-label ward-task performance of every representation, plotted as macro-F1 against bed-exit F1. The two metrics move together rather than trading off, and only the six-axis hand-crafted model reaches the upper-right region; every accelerometer-only representation, pretrained or fine-tuned, sits below it on both axes. These are the same values reported in Table 4 of the main text.

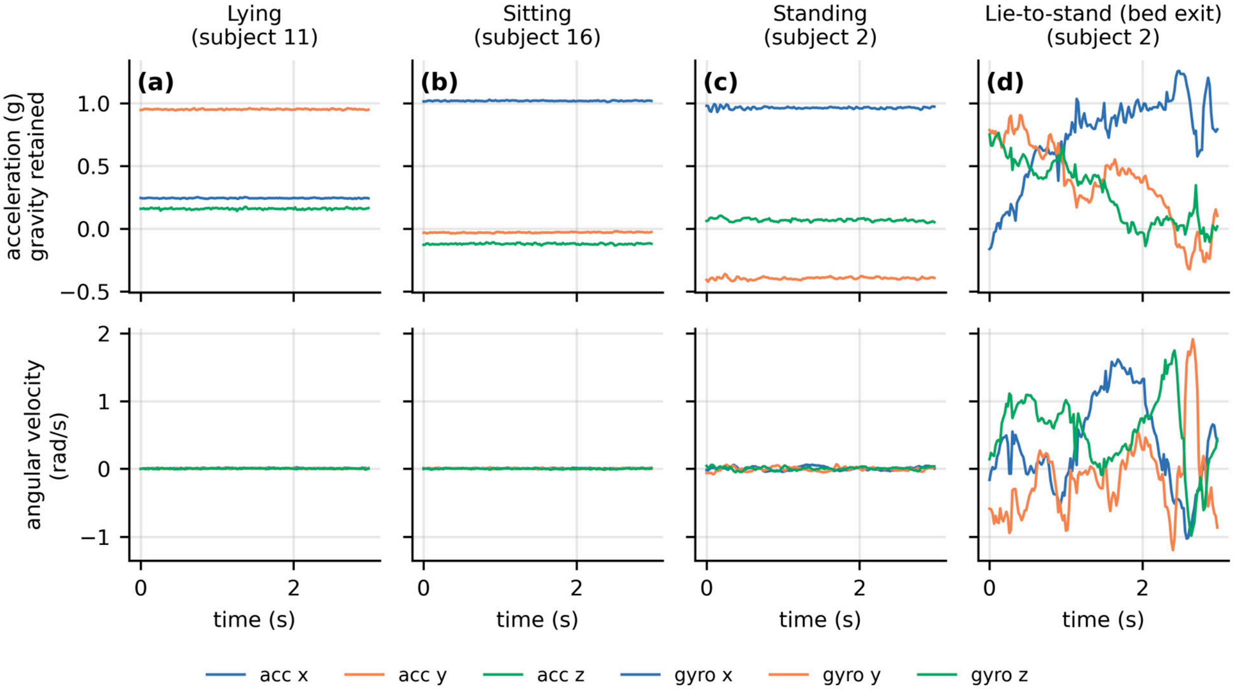

Figure S2. Raw six-axis signals as supplied by HAPT, with no filtering, detrending, or per-recording normalisation, for three static postures and one bed-exit transition (3 s excerpts; rows share a vertical scale): (a) lying; (b) sitting; (c) standing; (d) lie-to-stand. In each column the top panel is the accelerometer and the bottom panel the gyroscope. The accelerometer traces (top) sit at clearly different levels because the gravity vector splits differently across the axes with posture: gravity loads the y axis when lying (mean +0.95 g) and the x axis when sitting or standing (+1.02 g and +0.96 g), with sitting and standing separated mainly by the y component (-0.03 g versus -0.39 g). That offset is the posture signal, so filtering or per-recording normalisation would remove exactly what the ward task depends on, which is why no preprocessing is applied. The gyroscope traces (bottom) stay near zero while a posture is held (peak magnitude 0.02-0.08 rad/s) and swing only during the transition (1.91 rad/s), the complementary half of the same argument.

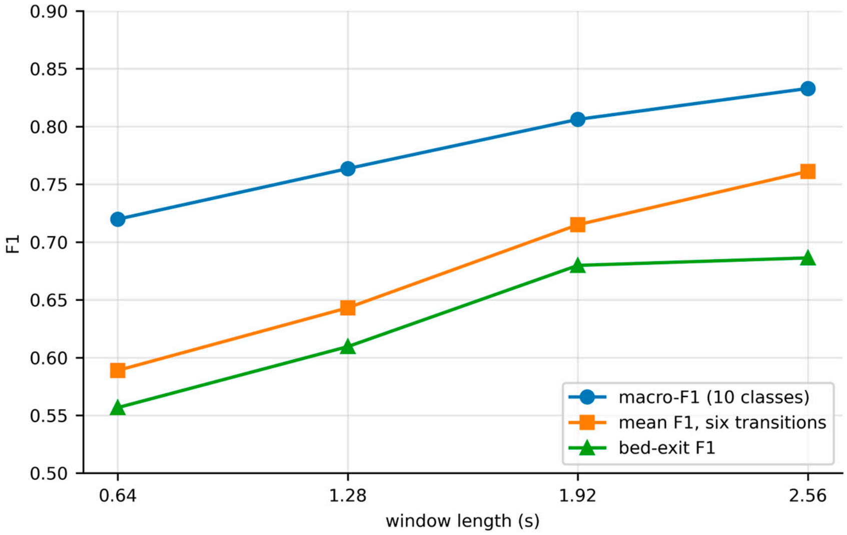

Figure S3. Recognition against analysis-window length, computed on the segments long enough to yield a window at every length so that the comparison is not confounded by survivorship. Longer windows help the transition classes most. These are the values tabulated in Table 3 of the main text; the figure was moved here at revision because it duplicated that table.

https://doi.org/10.3390/s26196073
